# How does imported pork regulate the supply and demand of China's pig market during the epidemic?—based on the analysis of African swine fever and COVID-19

**DOI:** 10.3389/fvets.2022.1028460

**Published:** 2022-11-24

**Authors:** Jingjing Wang, Gangyi Wang, Yiniu Cui, Jie Zhang

**Affiliations:** ^1^College of Economics and Management, Northeast Agricultural University, Harbin, China; ^2^Department of Marketing, College of Economics and Management, Northeast Agricultural University, Harbin, China; ^3^Chongqing Rural Revitalization Institute, Chongqing, China; ^4^School of Economics, Yunnan University, Kunming, China; ^5^Department of Agricultural Economics and Management, College of Economics and Management, Northeast Agricultural University, Harbin, China

**Keywords:** African swine fever, COVID-19, pork imports, pork prices, substitution effect

## Abstract

The pig industry is primarily a domestic industry in China is focused on ensuring the domestic pork supply. This paper analyzed changes in Chinese pork imports following the outbreaks of African Swine Fever (ASF) and COVID-19 between January 2017 to November 2020 and evaluated the impact of imported pork on the development of the swine industry in China. The results demonstrated that the shortage of domestic pork supply changed the import volume. ASF transformed imported pork from a complementary product to meet the diversified needs of domestic consumers into a critical substitute required to fill the supply gap. Following the COVID-19 outbreak, the substitution effect of imported pork decreased. ASF, has caused the supply capacity of pork in China to decrease, the price of pork to increase, leading to increased pork import in January 2019. At the end of 2019, pig slaughter decreased, while China cut tariffs on imported pork. The COVID-19 outbreak did not reduce China's pork imports in China, which declined after the global COVID-19 outbreak. Imported pork has made up for the supply gap during COVID-19, not impacting the level of production of the swine industry in China.

## Introduction

African Swine Fever (ASF) epidemic has had a massive impact on the pig industry in China. It is a virulent infectious disease with a mortality rate of up to 100% (1), the number of pigs culled nationwide reached 800,000 in China in 2018^1^. Many farmers' enthusiasm for replenishment has diminished, while some farmers even quit the pig industry. In October 2019, the breeding stocks of breeding sows and pigs decreased by 38.9 and 41.4%^2^ respectively from the same month of last year, indicating a shortfall in pork supply. Affected by the relationship between supply and demand, Chinese pork prices fluctuated irregularly, and seasonal changes were different from previous years. From August 2018 to February 2019, pork prices in China did not change much, and the Mid-Autumn Festival and National Day did not cause an increase in consumption as in previous years. From March 2019, pork prices began to increase, with the seasonal trends reversing of earlier years. In November, pork prices increased to 47.11 yuan/kg^3^, with a year-on-year increase of 300%. The import of chilled, fresh, and frozen pork^4^ has gradually increased. In 2019, the import volume of pork was close to 2 million tons. Meanwhile, 20 new countries for pork products have been added to ease the domestic supply tension. The outbreak of COVID-19 reduced pork supply and demand, slowing down the recovery of pig production capacity, and further increasing the gap in pork supply. The pork prices rose to nearly 50 yuan/kg^5^ in February 2020, reaching the peak since the outbreak of ASF. China still maintains a high volume of pork imports, which increased to 3.87 million tons in the first 11 months of 2020, nearly doubling from 2019.

The overall scale of imported pork in China is relatively small, and the domestic pork price is an essential factor affecting the import volume of pork. When there are no external shocks such as major epidemics, the proportion of imported pork in China is relatively stable. However, when external shocks such as significant epidemics occur, domestic pork prices are relatively high, and the widening of domestic and foreign price gaps leads to profitable imports. The proportion of pork imports will suddenly increase, and there will be “slight fluctuations,” namely the number of pork imports will suddenly increase in a short period, and it will drop again after the epidemic. For example, in 2008, the proportion of pork imports in China affected by PRRS rose from 0.2 in 2007 to 0.8%, and then dropped to 0.3%; in 2016, due to the superimposition of the pig cycle and environmental protection demolition factors, the ratio of imported pork increased from 2015. 1.4 rose to 2.6%, and then dropped to 2.2%; before the outbreak of ASF, the ratio of imported pork remained at around 2%. After the outbreak of ASF, the proportion of imported pork in China rose to 4.7% in 2019. According to the relationship between the year-on-year growth rate of the annual average price of live pigs in my country and the year-on-year growth rate of annual pork imports, it is found that the correlation coefficient between the two is as high as 0.82.

The “Double Epidemics”^6^ have caused pig market fluctuation in China and increased pork imports. Many previous studies have investigated how a disease outbreak can affect markets. Some studies have pointed out that a disease outbreak can affect pig production (2, 3). Meanwhile, disease outbreaks will distort the meat trade (4) and lead to biases in origin (5), and long-term trade bans will reduce supply (6). The impact of the disease on developed and developing countries is different (7). However, the impact on the export market was short-lived. The reopening of the market can quickly restore production, prices, and trade to their pre-outbreak levels (8).

The outbreak of ASF has led to increased pork prices and decreased demand (9). However, compared with diseases such as foot and mouth disease (FMD), ASF has a lower transmission potential, which is less affected by changes in the production structure (10). Inter-country pig trade can spread ASF (11, 12). Some scholars have pointed out that the impact of ASF on pork trade is more significant than that on production (13), but the current studies on ASF mainly focus on the effect of export. Export losses are the driving force behind the total costs of the epidemics (14). After the COVID-19 outbreak, some scholars have pointed out that the COVID-19 epidemic has disrupted the pork supply chain to some extent (3). However, the pig industry will continue to develop after adapting to market changes (15).

Armington^7^ model is mainly used to measure the substitution elasticity of imported goods and domestic goods (16, 17). It can be used to estimate the impact of a disease on trade. Many preliminary studies on how a disease affects the import demand, such as the influences of avian influenza on poultry trade (18), and the impact of mad cow disease on demand for Japanese beef (19). The Armington model they have used may serve as good pattern for the model used in this paper.

Generally speaking, existing studies rarely explore the impact of changes in imports on the pig industry. These studies pay less attention to changes in the substitution relationship between Chinese pork and imported pork under the impact of emergencies such as the “Double Epidemics.” How do these changes affect pig market supply also attracts little attention. Therefore, this paper uses the threshold model and the breakpoint regression model to measure the impact of the “Double Epidemics” on pork prices and imports in China by drawing on existing research. By measuring Armington substitution elasticity between Chinese pork and imported pork, this paper analyzed the changes in the import substitution value of Chinese pork pre- and post- “Double Epidemics,” and finds out why the increase in pork imports in China increased since the outbreak of “Double Epidemics.” This paper aims to answer the following two questions: First of all, the reasons for the changes in Chinese pork imports under the “Double Epidemics”? Secondly, how does the change in pork import volume stabilize the pork supply in the domestic market? This study can provide an objective basis for better restoring of pork supply and seeking long-term development of the pork trade in China.

## Chinese pork import situation

### Development trend of Chinese pork trade under the impact of “Double Epidemics”

**Figure 1:**
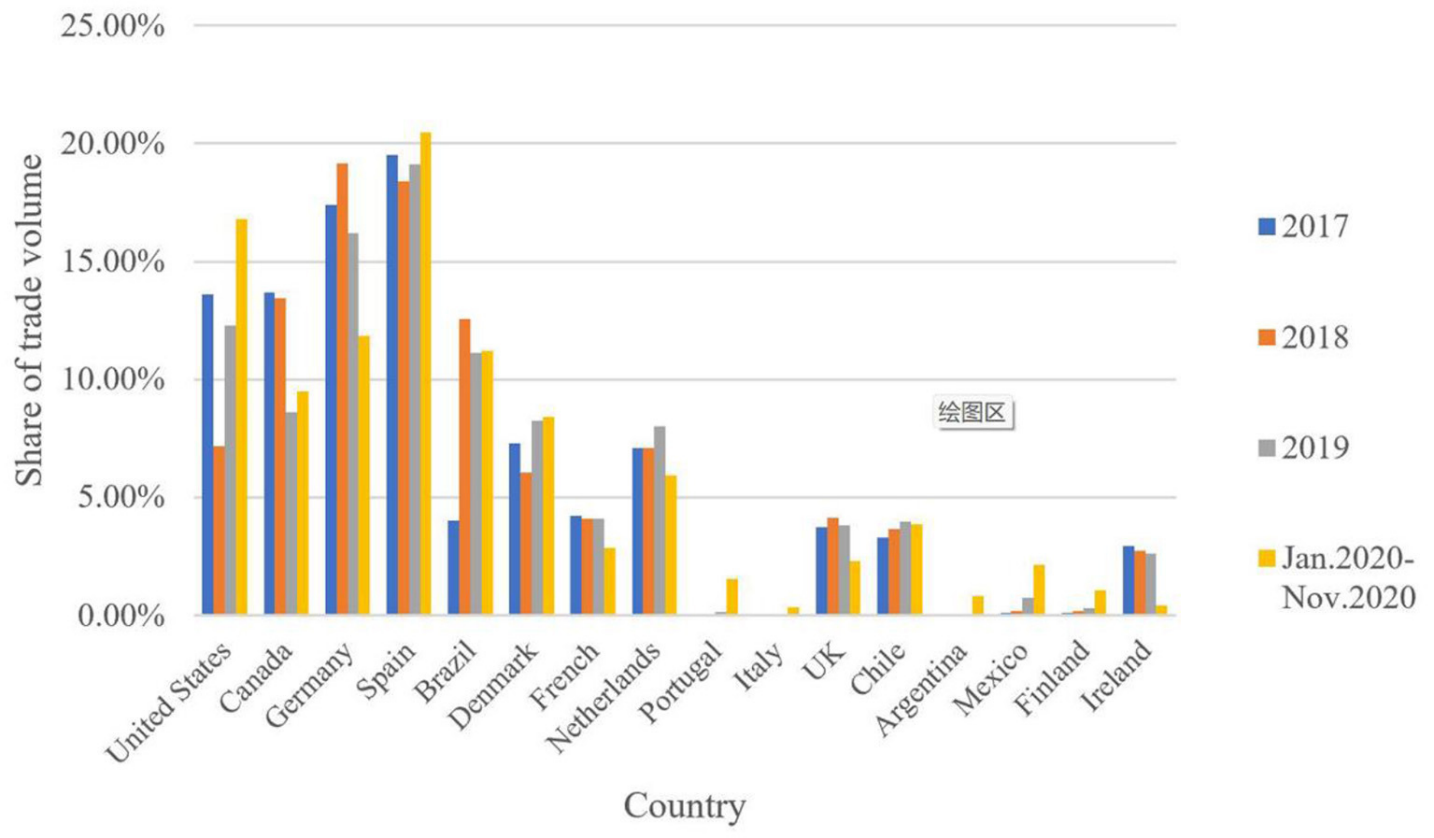
Evolution of Chinese pork imports from its trading partners from 2017 to 2020. Data from UN Comtrade.

After the outbreak of “Double Epidemics,” Chinese pork imports have become more widely sourced. In 2020, Chinese pork imports from Europe accounted for 55.8% ^8^of total pork imports, followed by North America (28.4%) and South America (15.8%) (Figure 1). In terms of regional distribution, since the outbreak of ASF, pork import trade between China and Europe has not increased significantly. After the outbreak of COVID-19, the proportion of imported pork has declined, but it is still the most crucial pork import area. In November 2020, the Chinese trade volume of imported European pork was 185,000 tons, accounting for 56% of the total import volume that month. A country-by-country analysis found that the proportion of Chinese pork imports from Germany, France, the Netherlands, and other countries declined after the outbreak of ASF.

In contrast, the proportion of pork imports from Denmark, Finland, and other countries increased. After the outbreak of COVID-19, the trade volume of imported pork from Italy and Portugal increased. In North America, Chinese imports of Canadian pork as a proportion of total pork imports have declined, while the proportion of imports of Mexican pork has increased; after the outbreak of COVID-19, the proportion of Chinese imports of pork from the United States has gradually increased.

South America has grown into another central pork import region. From 2017 to 2020, Chinese pork imports from South America increased by more than 8%. In November 2020, pork imports accounted for 18.3% of the total pork imports. With the outbreak of ASF, Chinese pork imports from Brazil have increased significantly, and it accounted for 14% of the total pork imports in November 2020. the number of pork imports from Argentina has increased after the outbreak of COVID-19. The amount of imported Argentine pork was 0.6% of the total import volume in November 2020.

### Development trend of Chinese pork import prices under the impact of “Double Epidemics”

For a long time, the import price of pork has been an advantage. Under the impact of the ”Double Epidemic,” the pork import prices in China have gradually increased, and the price gap between Chinese pork and imported pork has increased significantly. The price gap between the two reached the highest after the outbreak of COVID-19.

**Figure 2:**
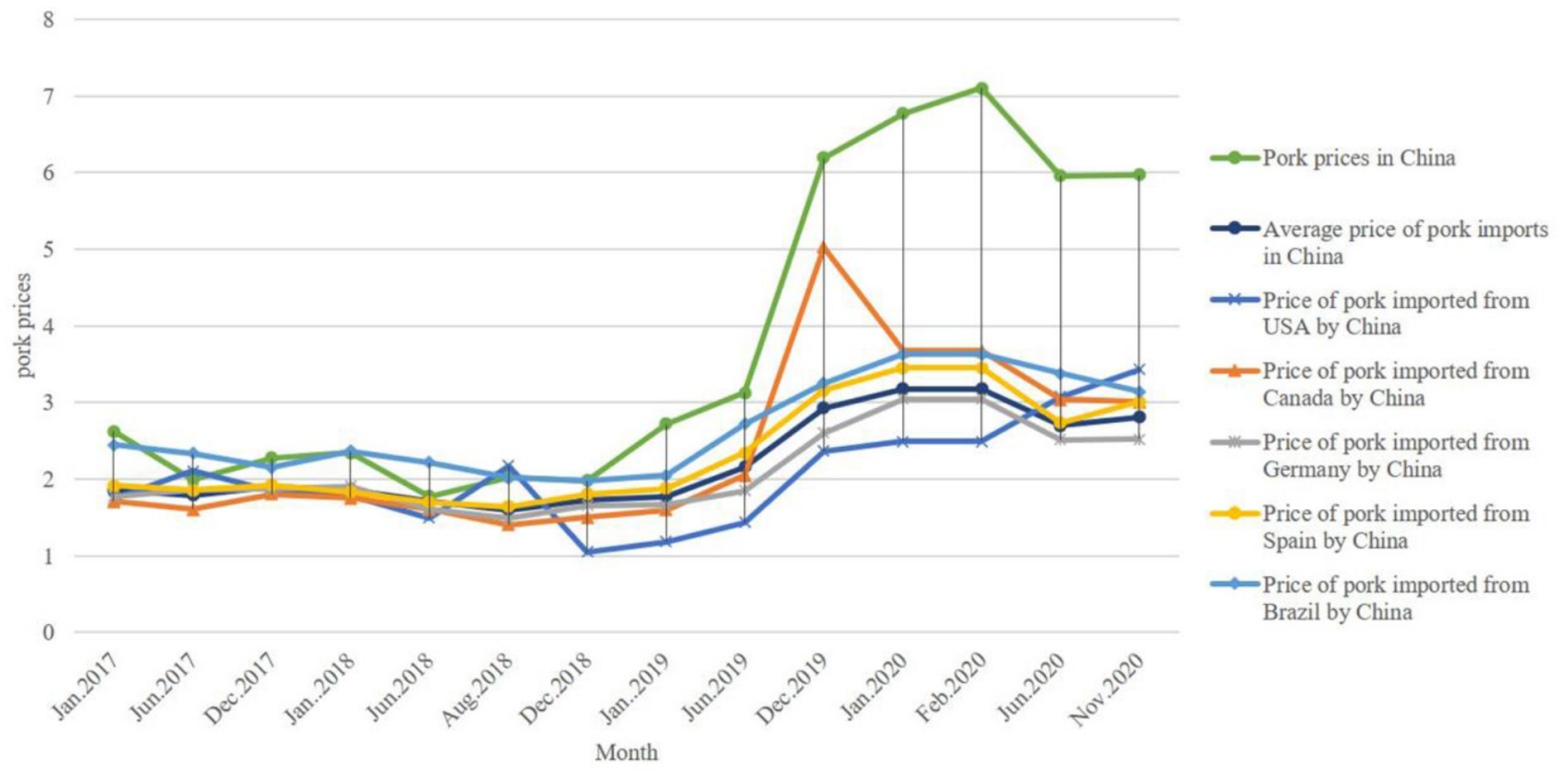
Pork prices of major importing countries and Chinese average pork import prices (USD/kg). Data from UN Comtrade.

As shown in Figure 2, except for a few years, pork prices in China are higher than imported prices. Before the outbreak of ASF, the price gap between the two was stabilized at < US$1, and imported pork did not show a significant price advantage. With the outbreak of ASF, pork prices in China have increased significantly, and the gap with imported prices has gradually increased. After the outbreak of COVID-19 in China, the price gap between them reached its highest in February 2020. After COVID-19 spread worldwide, the price gap turned into a downward trend.

After the outbreak of ASF, in terms of different countries, pork import prices in Germany, Spain, Canada, Brazil, and other countries have slowly increased. Still, pork import prices in the United States have fallen sharply. After the outbreak of COVID-19, pork import prices in Germany, Spain, and Canada have fallen, while pork import prices in the United States have increased. As the emerging pork import market of China, before the outbreak of ASF, the prices of pork imported from Brazil were not significantly different from that in domestic. However, under the “Double Epidemics” continuous impact, the price of pork imported from Brazil has gradually decreased.

## Materials and methods

### Model design

#### Armington model

Armington model was used to test the substitution effect of Chinese pork imports under the impact of the “Double Epidemics.” Armington (20) believed that products from different countries or regions are not perfectly substituted from the perspective of either the demand side or the supply side. Armingtonhas used the constant elasticity of substitution (CES) form for the representative subutility function for an industry (21), namely, Armington elasticity of substitution. Armington elasticity reflects the degree of substitution between domestic and imported goods. Without considering the impact of policies, the degree of substitution between domestic and imported goods is determined mainly by relative price changes. If domestic and imported products are highly substitutable, then price changes will cause higher demand changes. Therefore, this paper constructed the Armington model to test the “Double Epidemics” impact on pork imports. The specific derivation of the model is as follows:

Assuming that the total demand level is *Y*_*i*_, the function of maximizing the utility of a specific commodity in the i country can be as follows:


(1) $${\text{MaxU}}_{\text{i}}=[\delta_{i}D_{i}^{\frac{\sigma_{i}-1}{\sigma_{i}}}+(1-\delta_{i})M_{i}^{\frac{\sigma_{i}-1}{\sigma_{i}}}){]}^{\frac{\sigma_{i}}{\sigma_{i}-1}}$$

(2) $${\text{ Y}}_{\text{i}}{=P}_{i}^{D}D_{i}{+P}_{i}^{M}M_{i}$$

Among them, U_i_ is the combination utility of the same domestic and imported products consumed by consumers in the i country, D_i_ represents the quantity of the domestic goods consumed by the i country, M_i_ is the quantity of imported goods consumed, and ${\text{P}}_{\text{i}}^{\text{D}}$ indicates the domestic production price of the product in the i country, ${\text{P}}_{\text{i}}^{\text{M}}$ indicates the import market price of the product in the i country. δ_*i*_ denotes the preference coefficient of the product, and σ_*i*_ are the constant substitution elasticity of the product and the imported product in the i country; σ_*i*_ ϵ[(0,1)U(1,+ ∞)],δ_*i*_i, *D*_*i*_, *M*_*i*_ are all positive. According to the condition of maximizing consumer utility, that is, the marginal rate of substitution of two commodities is equal to the ratio of their prices, further derivation can be obtained as:


(3) $$\begin{array}{l} \frac{{\text{M}}_{\text{i}}}{{\text{D}}_{\text{i}}}={\left(\frac{{\text{P}}_{\text{i}}^{\text{D}}}{{\text{P}}_{\text{i}}^{\text{M}}}\frac{1-\delta_{\text{i}}}{\delta_{\text{i}}}\right)}^{\sigma_{\text{i}}} \end{array}$$

Taking the natural logarithms on both sides, the following equation can be obtained:


(4) $$\begin{array}{l} \text{ln}\frac{{\text{M}}_{\text{i}}}{{\text{D}}_{\text{i}}}=\sigma_{\text{i}}\text{ ln}\frac{1-\delta_{\text{i}}}{\delta_{\text{i}}}+\sigma_{\text{i}}\text{ ln}\frac{{\text{P}}_{\text{i}}^{\text{D}}}{{\text{P}}_{\text{i}}^{\text{M}}} \end{array}$$

Let $Y=\text{ln}\frac{{\text{M}}_{\text{i}}}{{\text{D}}_{\text{i}}},\text{ X}=\text{ln}\frac{1-\delta_{\text{i}}}{\delta_{\text{i}}},\gamma =\sigma_{\text{i}}\text{ ln}\frac{{\text{P}}_{\text{i}}^{\text{D}}}{{\text{P}}_{\text{i}}^{\text{M}}},\varphi =\sigma_{\text{i}},$ Simplified to


(5) $$\begin{array}{l} \text{Y}=\gamma +\varphi \text{X} \end{array}$$

In summary, this paper constructed eight models to test the impact of relative prices in the current period and the lagging period on Chinese pork import demand before and after the outbreak of the “Double Epidemics.” By comparing the differences in the estimated value changes before and after the outbreak, consumers' preferences for pork demand and the degree of substitution elasticity under the impact of the “Double Epidemics” can be estimated as follows:


(6) $$\begin{array}{l} {\text{Y}}_{\text{it}}=\gamma +\varphi {\text{X}}_{\text{it}}+\varepsilon_{\text{it}} \end{array}$$

(7) $$\begin{array}{l} {\text{Y}}_{\text{it}}=\gamma +\varphi {\text{X}}_{\text{it}-1}+\varepsilon_{\text{it}} \end{array}$$

X_it_ and X_it−1_ represent the relative price of the current period and the lagging period. According to the derivation principle of the model, elasticity substitution *φ* is expressed as the sensitivity of a commodity's import demand to changes in the domestic price of the commodity. It is assumed that there is a substitution relationship between imported goods and domestic goods. In that case, the relative price increase of domestic goods will lead to a relative increase in imported goods. At this time, Y and X change in the same direction, and the sign of *φ* should be positive. It is assumed that there is a complementary relationship between imported goods and domestic goods. In that case, an increase in the relative price of domestic goods will lead to a relative decrease in the number of imported goods. At this time, Y and X change in opposite directions, and the sign of *φ* should be negative. In addition, if domestic consumers have significant preferences for pork from different import source countries, the intercept term *γ* will change.

#### Threshold model

It is concluded that there may be a non-linear relationship between the “Double Epidemics” on pork prices in China, and there may be a tipping point for pork prices in China. As an effective method to identify and test the critical points in the system, the threshold model can effectively solve this problem. Therefore, according to the threshold test method proposed by Hansen (22), we used Threshold regression model to observe the phased impact of the “Double Epidemics” outbreak on pork prices in China. The setting of the model is as follows:


(8) $$\begin{array}{l} {\text{Price}}_{\text{it}}=\omega_{0}+\omega_{1}\times {\text{K}}_{\text{it}}+\omega_{2}\times {\text{ASF}}_{\text{t}}\times \text{f }\left(\text{q}\leq {\text{month}}_{1}\right)\\ \;+\omega_{3}\times {\text{ASF}}_{\text{t}}\times \text{f }\left({\text{month}}_{1}<\text{q}\leq {\text{month}}_{2}\right)+\omega_{4}\times {\text{ASF}}_{\text{t}}\\ \;\times \text{f }\left(\text{q}>{\text{month}}_{2}\right)+\theta_{\text{it}} \end{array}$$

(9) $$\begin{array}{l} {\text{Price}}_{\text{it}}=\omega_{5}+\omega_{6}\times {\text{K}}_{\text{it}}+\omega_{6}\times {\text{COVID}}_{\text{t}}\times \text{f }\left(\text{q}\leq {\text{month}}_{3}\right)\\ \;+\omega_{7}\times {\text{COVID}}_{\text{t}}\times \text{f }\left({\text{month}}_{3}<\text{q}\leq {\text{month}}_{4}\right)+\omega_{\text{8}}\\ \;\times {\text{COVID}}_{\text{t}}\times \text{f }\left(\text{q}>{\text{month}}_{4}\right)+\theta_{\text{it}} \end{array}$$

Where *Price*_it_ is the pork price in China at time t, core explanatory variable ASF_t_ represents the impact of ASF in China at time t, and COVID_t_ represents the impact of COVID-19 at time t. Moreover, *K*_*it*_ represents the control variable, ω_0_, ω_1_…ω_8_ are estimated coefficients, and θ_it_represents the residual term.

#### Breakpoint model

Breakpoint was used regression to test the relationship between the changes in Chinese pork prices and import volume under the outbreak of “Double Epidemics.” The breakpoint regression is a random experimental model, which can more effectively avoid the endogeneity problem caused by parameter estimation, resulting in more explicit causal inference. According to the certainty of the impact on the individual at the discontinuity point, breakpoint regression methods can be divided into two categories: one type is accurate breakpoint regression, and the other is fuzzy breakpoint regression. Sharp regression discontinuity (SRD) means that the relationship between the individual and the treatment effect is determined, and when the breakpoint X = m, the probability of an individual being processed jumps from 0 to 1. Fuzzy regression discontinuity (FRD) means that the relationship between individual and treatment effect is random. At the breakpoint X = m, the probability of the individual being processed jumps from a to b, and satisfies the law of 0<a<b<1. As the outbreak time of ASF and COVID-19 in China was fixed, namely August 2018 and January 2020, respectively, which met the condition of the exact breakpoint. Therefore, this paper adopts Sharp regression discontinuity and builds models to test the impact of the “Double Epidemics” on the prices and import volume of pork in China as:


(10) $${\text{import}}_{\text{it}}=\alpha_{1}+\beta_{1}\times {\text{h}}_{\text{it}}+\vartheta_{\text{it}}$$

(11) $${\text{Price}}_{\text{it}}=\alpha_{2}+\beta_{2}\times {\text{d}}_{\text{it}}+\beta_{3}{\text{G}}_{\text{it}}+\tau_{\text{it}}$$

Where i denotes a country, t represents the period; Price_it_ and import_it_ are the outcome variables, Price_it_ is the pork price in China at time t; import_it_ represents the growth rate of pork imports between China and country i; h_it_, d_it_ are taken as the processing variables; *G*_*it*_ is the concomitant variable; τ_it_, ϑ_it_ are the residual items; *α*_1_, *α*_2_, *β*_1_, *β*_2_, *β*_3_ are estimated coefficients.

### Data

Based on the reliability and accuracy of data selection, this paper builds a panel model based on the monthly data of China and 13 pork-importing countries^9^, including the United States, Canada, and Germany, from January 2017 to November 2020. In 2020, Chinese pork imports from these 13 countries accounted for 97.9% ^10^of the total pork imports. And all the processing completes with stata15.0.

#### Armington model

Based on data availability, this paper chooses to express Chinese total pork production as the product of the slaughtered volume of pigs above the designated size (10,000 heads) and the production of primary products (kg/head). Since the output of main pig products in China has not changed much since 2017, this paper chooses to substitute the main product output of pigs in the ASF outbreak (122.79 kg/head) into the formula. At the same time, to enhance the stability and weaken the heteroscedasticity of the data, this paper chooses to take the natural logarithm of the following variables. The specific instructions are as Table 1.

**Table 1:** Meaning of primary variables and data sources.

**Variables**	**Meanings**	**Calculation**	**Data source**
*M*_*i*_	The volume of Chinese pork imports from country i	from China Customs Database	UN Comtrade
*D*_*i*_	Chinese pork demand based on the country i	(Chinese total pork imports)-(Chinese pork imports to the i source country of pork imports) + (Chinese total pork production)-(Chinese total pork exports)	General Administration of Customs, PRC; Ministry of Agriculture and Rural Affairs “Monthly Report on the Supply and Demand Situation of Agricultural Products”^a^ (bulk agricultural products)
$P_{i}^{M}$	Chinese pork wholesale price	from the website of the Ministry of Agriculture and Rural Affairs	Ministry of Agriculture and Rural Affairs of China
$P_{i}^{D}$	pork price of importing country i	pork imports amount/pork import volume	UN Comtrade

a For example, the Nov. 2020 report comes from http://www.moa.gov.cn/ztzl/nybrl/rlxx/202012/t20201222_6358612.htm which is reported by the Ministry of Agriculture and Rural Affairs of Chin.

#### Examination of the impact on Chinese pork prices under the “Double Epidemics”

(1) Explained variable: Taking January 2017 as the base period, deflate Chinese pork prices based on the CPI index^11^ published by the National Bureau of Statistics to eliminate inflationary factors, and treat the processed Chinese pork prices as the explained variable.

(2) The core explanatory variables in the threshold model are the impact of ASF and the impact of COVID-19. Among them, the impact of ASF is expressed as the ratio of the provinces with ASF in 29 provinces each month^12^ (except Tibet and Hebei provinces); The impact of COVID-19 is expressed as the monthly number of confirmed COVID-19 (per million people) in China and each trading partner country^13^.

In the breakpoint regression, different thresholds are set as treatment effect variables. To ensure the accuracy of the results, this paper considers pork prices in an importing country i (${\text{P}}_{\text{i}}^{\text{D}}$), the quantity of pork imported from China to country i (M_i_), the exchange rate (rate_i_), and seasonal factors^14^ are covariates. The data is selected from the database of UN Comtrade, the Ministry of Agriculture and Rural Affairs and the International Monetary Fund (IMF).

#### Examination of the changes in Chinese pork import volume under the “Double Epidemics”

(1) Explained variables and treatment variables: Taking the growth rate of pork import volume^15^ as the explained variable, the Month is set as the treatment variable^16^.

(2) Covariate: seasonal factors.

## Results

### Descriptive analysis

This paper used the panel data from January 2017 to November 2020 to construct the impact sample of ASF. At the same time, the outbreak time point of ASF (August 2018) was used as the new starting point to construct the COVID-19 impact sub-sample.

Armington was used to analyzing the causes of the change in Chinese pork import volume caused by the outbreak of “Double Epidemics.” The breakpoint regression model was used to calculate the impact of “Double Epidemics” on Chinese pork import volume and pork price change. Indicators include import relative demand, relative price, lagging relative price, a growth rate of pork import volume, Chinese pork price, Chinese pork import volume from the I country, pork import price, and exchange rate. Descriptive statistics of specific variables are shown in Table 2. Part A reports on the entire sample that the African outbreak has hit. Part B reports subsamples that the COVID-19 outbreak has hit. The growth rates of pork imports were significantly different, with a minimum value of −0.94, a maximum value of 84, and an average value of 0.32. The Chinese average price of pork is higher than the import price, and the standard deviation is also more significant than the import price, but the maximum value of the imported pork price is greater than the Chinese price. Compared with the whole sample, the COVID-19 epidemic impact sub-sample had a larger mean of import relative demand, and the Chinese price and import price of pork were higher.

**Table 2:** Descriptive statistical analysis.

**Model**	**Variable**		**Sample size**	**Mean**	**Standard deviation**	**Max**.	**Min**.
**A: Sample affected by the ASF epidemic**							
Armington model	The relative demand for imports	*Y*_*it*_	605	−5.86	1.62	−3.04	−12.16
	Relative price	*X*_*it*_	605	0.43	0.46	2.80	−2.07
	Lags a period relative prices	*X*_*it*−1_	594	0.42	0.46	2.80	−2.07
Breakpoint regression model 1	The growth rate of pork imports	*increase*_*i*_	592	0.32	3.65	84.00	−0.94
Threshold regression model	Impact of ASF	*ASF*_*t*_	611	−0.94	5.69	1.48	1
Breakpoint regression model 2	Chinese price of pork	$\text{ln }P_{i}^{M}$	611	1.15	0.51	1.96	0.49
	Chinese Pork Imports from Country i	ln *M*_*i*_	611	15.65	1.57	18.31	9.39
	The import price of pork	$\text{ln }P_{i}^{D}$	611	0.73	0.44	3.98	−0.88
	The exchange rate	ln *rate*_*i*_	611	1.68	2.12	7.06	0.03
**B: Sub–samples suffering from the impact of COVID−19**							
Armington model	The relative demand for imports	*Y*_*it*_	364	−5.52	1.51	−3.04	−10.81
	Relative price	*X*_*it*_	364	0.61	0.49	2.80	−2.07
	Lags a period relative prices	*X*_*it*−1_	364	0.58	0.49	2.80	−2.07
Threshold regression model	Impact of COVID−19	*COVID*_*t*_	143	2022.865	2922.703	13610.37	6.81
Breakpoint regression model 2	Chinese price of pork	$\text{ln }P_{i}^{M}$	364	1.44	0.48	1.96	0.66
	Chinese Pork Imports from Country i	ln *M*_*i*_	364	15.93	1.46	18.31	10.8
	The import price of pork	$\text{ln }P_{i}^{D}$	364	0.83	0.52	3.98	−0.88
	The exchange rate	ln *rate*_*i*_	364	1.65	2.11	6.97	0.03

### Test of Chinese pork import substitution effect under the “Double Epidemic”

The intercept terms of the eight models pass the test at the significance level of 1 %. It shows significant differences in Chinese consumers' preferences for pork from different importing countries in the pre- and post- “Double Epidemics” periods.

Before the outbreak of ASF, Chinese pork and imported pork were complementary. The substitution elasticity of Armington passed the test at the significance level of 1%, indicating that the consumption of imported pork decreased by 2.67% when the pork price in China increased by 1%. For every 1% increase in pork prices with a lag period, the consumption of imported pork decreased by 2.402%. Before the outbreak of ASF, the Chinese pork self-sufficiency rate was sufficient, the supply was stable, and the consumer demand was diverse. Most imported pork was used to meet the diversified needs of consumers, and the relative price rise of Chinese pork and imported pork would not increase the import demand.

After the outbreak of ASF, the relationship between Chinese pork and imported pork has become a substitute. For every 1% increase in pork price in China, the consumption of imported pork increases by 0.84%, and for every 1% increase in the lagging pork price, the consumption of imported pork increases by 0.6%. After the outbreak of ASF, the number of pigs in China declined, the price of pork rose, and the implementation of the China Pig Transfer Policy enlarged the pork supply gap in some regions. Pork import became an essential way of pork supply after the outbreak of ASF (Table 3).

**Table 3:** Comparison of Armington model estimates in the pre– and post– ASF epidemics periods.

**Variable name**	**Pre**				**Post**			
	**Model 1**		**Model 2**		**Model 3**		**Model 4**	
	**Coef**.	**Z Value**	**Coef**.	**Z Value**	**Coef**.	**Z Value**	**Coef.e**	**Z Value**
cons.	−5.97***	−73.98	−5.99***	−70.99	−6.02***	−45.41	−5.84***	−40.97
*X*_*it*_	−2.67***	−5.61	–	–	0.84***	4.89	–	–
*X*_*it*−1_	–	–	−2.40***	−4.65	–	–	0.60**	3.28
N	241		228		364		351	

^***^, ^**^, and ^*^ indicate significance at the level of 1%, 5% and 10%, respectively.

After the outbreak of COVID-19, the import substitution elasticity of pork in China decreased slightly, but there was still a significant substitution relationship. For every 1% increase in pork price in China, residents' consumption of imported pork will increase by 0.796%. For every 1% increase in the lagging pork price, the consumption of imported pork will increase by 0.5%. After the outbreak of COVID-19, the slaughtering, processing, and distribution phases of the meat supply chain of Chinese trading partners has drastically affected (11, 15). It reduced the pork trade with China (Table 4).

**Table 4:** Comparison of Armington model estimates in the pre– and post– COVID−19 epidemics periods.

**Variable name**	**Pre**				**Post**			
	**Model 5**		**Model 6**		**Model 7**		**Model 8**	
	**Coef**.	**Z Value**	**Coef**.	**Z Value**	**Coef**.	**Z Value**	**Coef**.	**Z Value**
cons.	−6.00***	−28.96	−5.88***	−27.77	−5.42^***^	−58.55	−5.14***	−49.80
*X*_*it*_	0.078	0.20	–	–	0.796***	7.72	–	–
*X*_*it*−1_	–	–	−0.08	−0.21	–	–	0.50***	4.31
N	221		208		143		130	

^***^, ^**^, and ^*^ indicate significance at the level of 1%, 5% and 10%, respectively.

It is assumed that the elasticity of import substitution of a commodity tends to 1. In that case, it means that the change in the international price of the commodity will be passed on to the import volume of the commodity to a large extent and will not have a significant impact on the domestic economy (23). After the outbreak of ASF, the Armington elasticity of substitution of Chinese pork is 0.837. Even after the COVID-19 outbreak, the substitution elasticity of Armington was 0.796, indicating that the increase in pork imports has not yet threatened the Chinese pig industry.

### Test of changes in Chinese pork imports under the “Double Epidemic”

The results show that in the month of the outbreak of ASF (August 2018), there was no obvious breakpoint in the growth rate of China's pork imports. To solve the problem of the spread of epidemic diseases caused by the transportation of live pigs, the Chinese government proposed to change the mode of transportation of live pigs at the end of 2019, from “transporting pigs” to “transporting meat.” There is a severe imbalance between pork supply and demand, and the pork price difference between production and sales areas has increased. With the arrival of the peak season of domestic pork consumption, the growth rate of pork imports in China volume has made a positive “jump” in January 2019, and bandwidth tests are significant, which means that the ASF epidemic has had a lagging impact on changes in pork imports in China, driving a substantial increase in imports (Table 5).

**Table 5:** Breakpoint regression results of “Double Epidemic” on the growth rate of Chinese pork imports (controlling seasonal variables).

**Model**	**Lwald100**		
	**Coef**.	**Standard error**	***P*-Value**
Dec.2018	0.33	0.24	0.167
Jan.2019	2.29^***^	0.42	0.00
Jan.2020	4.44*	2.48	0.07

^***^, ^**^, and ^*^ indicate significance at the level of 1%, 5% and 10%, respectively.

In January 2020, after the outbreak of the domestic COVID-19 epidemic, the growth rate of pork imports showed a significant upward “jump” in January 2020. The reason is that China has once again lowered tentative tariffs on pork imports. Import costs are directly related to ingtrading policies. While domestic pork prices were rising, pig slaughter in China reached a trough at the end of 2019, and the tariff changes drove a sharp increase in pork imports. Therefore, after the outbreak of COVID-19, the growth rate of pork imports did not have a downward breakpoint.

### Test of the impact of the “Double Epidemic” on pork prices in China

#### Characteristics of threshold test of Chinese pork price

Table 6 shows that under the impact of ASF, pork prices in China can be divided into three stages, with December 2018 and August 2019 as the boundary, respectively. Before December 2018, the impact of the ASF shock on price was negative, with an estimated coefficient of −0.33. Between December 2018 and August 2019, the impact of ASF shocks on domestic prices changed, with the estimated coefficient changing to 0.2. When the threshold is August 2019, the impact of ASF shocks on domestic prices increases, with the estimated coefficient increasing to 0.66.

**Table 6:** Threshold effect test of “double epidemic” on domestic pork price changes.

**Model**	**Variable**	**Coef**.	**SE**	***P*-Value**
Threshold effect of ASF	$\text{ln }P_{i}^{D}$	0.03**	0.01	0.02
	*lnM*_*i*_	−0.004	0.003	0.19
	covid	0.00**	0.00	0.03
	ASF (Month ≤ 201812)	−0.33^***^	0.05	0.00
	ASF (201812 < Month ≤ 201908)	0.02	0.05	0.71
	ASF (Month>201908)	0.66***	0.05	0.00
Threshold effect of COVID−19	$\text{ln }P_{i}^{D}$	−0.02	0.02	0.36
	*lnM*_*i*_	−0.04*	0.02	0.05
	ASF	0.09	0.09	0.31
	COVID (Month ≤ 202006)	−0.00002***	0.00	0.00
	COVID (202006 < Month ≤ 2020009)	0.00002***	0.00	0.00
	COVID (Month>202009)	−0.000007***	0.00	0.00

^***^, ^**^, and ^*^ indicate significance at the level of 1%, 5% and 10%, respectively.

The supply of live pig market this year will be affected by the stock of breeding pigs in the previous year. From September to December 2017, the stock of breeding sows in China decreased gradually due to environmental protection policies, which affected the number of live pigs in the second half of 2018. At the beginning of the ASF epidemic, the number of pigs slaughtered and stocked in China increased to different degrees (Table 7). The number of pigs slaughtered increased significantly while the stock increased slightly, so the pork price decreased. Pork prices increased after the live pig stock began to decline in December 2018. Still, the impact of ASF on prices was not significant due to the increase in slaughter volumes, and a significant increase in pork prices occurred after a significant decline in slaughter volumes in August 2019.

**Table 7:** The stock of breeding sows, the stock of pigs, the number of slaughtered pigs, and their respective sequential growth rates in China (unit: 10,000 head, %).

**Month**	**Breeding sows**		**Pigs**		**Slaughtered pigs**	
	**Stock**	**Sequential growth rates**	**Stock**	**Sequential growth rates**	**Number**	**Sequential growth rates**
Jan.2017	3717.82	–	35556.5	–	2075.44	–
Mar.2017	3699.23	−0.50	35840.3	0.80	1719.18	−17.17
Jun.2017	3651.29	−1.30	35373	−1.30	1774.96	3.24
Sep.2017	3560.76	−2.48	34879.8	−1.39	1871.13	5.42
Dec.2017	3486.43	−2.09	34045	−2.39	2324.54	24.23
Mar.2018	3676.88	5.46	35694.1	4.84	1917	−17.53
Jun.2018	3488.82	−5.11	34318.9	−3.85	1957.83	2.13
Sep.2018	3374.73	−3.27	34213.8	−0.31	1923	−1.78
Oct.2018	3334.23	−1.20	34248	0.10	1950.95	1.45
Nov.2018	3290.89	−1.30	34008.3	−0.70	2006.51	2.85
Dec.2018	3215.2	−2.30	32750	−3.70	2288.35	14.05
Mar.2019	2877.95	−10.49	28864.9	−11.86	1855.72	−18.91
Jun.2019	2556.4	−11.17	25481.3	−11.72	1758.24	−5.25
Sep.2019	2057.68	−19.51	20198.9	−20.73	1233.72	−29.83
Dec.2019	2200.19	6.93	20530.4	1.64	1452.77	17.76
Mar.2020	2327.85	5.80	22208.5	8.17	1171.3	−19.37
Jun.2020	2646.18	13.67	25230.7	13.61	1327.1	13.30
Sep.2020	2933.79	10.87	28819.6	14.22	1285.2	−3.16
Dec.2020	3164.8	7.87	31979	10.96	2060.35	60.31

COVID-19 has a double threshold effect on pork prices in China. The threshold values were in June 2020 and September 2020, respectively. After the outbreak of COVID-19, the fluctuation of domestic pork prices decreased and experienced a process of first decreasing, then increasing, and then decreasing. Before June 2020, the impact of COVID-19 on domestic pork prices was negative. When the threshold was between June 2020 and September 2020, the coefficient became positive. The effect of COVID-19 on Chinese pork prices has changed and produced a significant promoting effect. After September 2020, the inhibitory effect of COVID-19 on domestic pork prices increased, and the coefficient was negative. As higher pork prices will increase the stock of breeding sows, with the dual drive of import regulation and policy incentives, the stock of breeding sows and pig stock in China will gradually increase at the end of 2019, and the pig production capacity will gradually recover. In June 2020, the slaughter volume increased slightly due to the increase in domestic pork prices. However, as pig stock and slaughter volume in China returned to normal, the domestic pork price decreased again.

#### Changes in Chinese pork prices at different threshold stages

**Figure 3:**
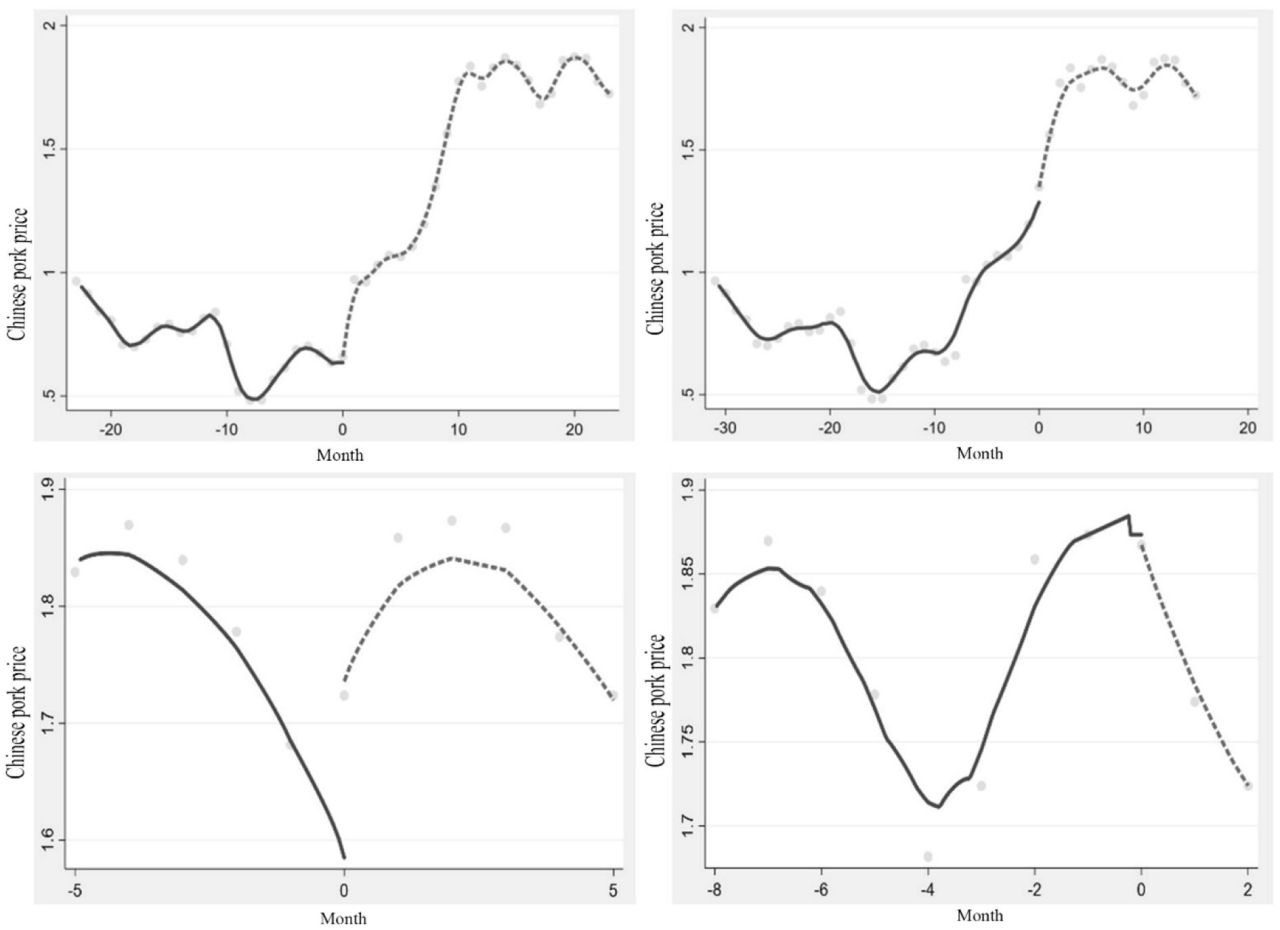
Breakpoint regression results of different thresholds on Chinese pork price.

The threshold regression results show that the impact of Chinese pork price varies in stages with the change of the impact degree of “Double Epidemic.” Subsequently, is there a jump in the Chinese pork price level? Therefore, the different thresholds obtained were estimated as breakpoints, and the results showed a breakpoint in the impact of the “Double Epidemic” on Chinese pork prices. The Chinese pork price had a significant upward breakpoint in December 2018, followed by a more significant upward breakpoint in August 2019. The Chinese pork price dramatically jumped at the breakpoint in June 2020 and jumped downward in September (Table 8, Figure 3).

**Table 8:** Test results of adding covariables.

**Model**	**Lwald100**			**Lwald200**			**Lwald400**		
	**Coef**.	**Standard error**	***P*-Value**	**Coef**.	**Standard error**	***P*-Value**	**Coef**.	**Standard error**	***P*-Value**
Dec.2018	0.02***	0.00	0.00	0.06***	0.0005	0.00	0.04***	0.01	0.00
Aug.2019	0.06***	0.00	0.00	0.17***	0.01	0.00	0.27***	0.03	0.00
Jun.2020	0.15***	0.004	0.00	0.17***	0.02	0.00	0.17***	0.02	0.00
Sep.2020	−0.01***	0.00	0.00	−0.08***	0.007	0.00	−0.02**	0.01	0.01

^***^, ^**^, and ^*^ indicate significance at the level of 1%, 5% and 10%, respectively.

#### Further consider the role of import and seasonal factors

After controlling for import and seasonal factors, the results showed that no matter how the bandwidth is, the coefficient and significance of the jump value generated by pork prices in China do not change significantly. The “jump” of 4 covariables at the breaking point under different bandwidths was tested. It was found that in different threshold models, the test results of the three covariables at the breaking point were insignificant, such as pork price of importing country i, the quantity of imported pork in importing country i, and the exchange rate. It can be judged that the Chinese pork price “jump” is related to the season but has nothing to do with the change in import (Table 9).

**Table 9:** Covariate breakpoint regression test results.

**Variable**	**Bandwidth**	**201812**		**201908**		**202006**		**202009**	
		**Coef**.	***P*-Value**	**Coef**.	***P*-Value**	**Coef**.	***P*-Value**	**Coef**.	***P*-Value**
$\text{ln }P_{i}^{D}$	lwald100	−0.044	0.6	−0.10	0.30	0.04	0.87	0.04	0.77
ln *rate*_*i*_		0.004	0.996	0.01	0.993	0.01	0.99	−0.001	0.99
ln *M*_*i*_		−0.06	0.915	−0.31	0.717	0.21	0.79	0.02	0.96
Season		0	omitted	0	omitted	0	omitted	0	omitted
$\text{ln }P_{i}^{D}$	lwald200	−0.15	0.28	−0.12	0.05	0.17	0.28	0.11	0.57
ln *rate*_*i*_		0.24	0.86	−0.0006	0.99	0.01	0.99	−0.004	0.99
ln *M*_*i*_		−0.06	0.95	−0.27	0.57	−0.11	0.80	0.100	0.86
Season		0.10***	0.002	−0.04^***^	0.001	0.40***	0.001	0	omitted
$\text{ln }P_{i}^{D}$	lwald400	0.006	0.932	−0.06	0.23	0.17	0.30	0.07	0.64
ln *rate*_*i*_		0.12	0.88	0.07	0.99	0.01	0.99	−0.005	0.99
ln *M*_*i*_		−0.08	0.88	−0.40	0.25	−0.17	0.69	−0.09	0.83
Season		0.50***	0.001	0.11***	0.00	0.46***	0.00	0.02	0.001

^***^, ^**^, and ^*^ indicate significance at the level of 1%, 5% and 10%, respectively.

## Discussion

In normal years, pork supply in China mainly depends on domestic, and pork import has little impact on the supply. However, after the outbreak of the epidemic, imported pork can play a role in making up the supply and suppressing the domestic pork price, becoming one of the crucial means to alleviate the imbalance between supply and demand. The elasticity of substitution between Chinese pork and imported pork shows that the substantial increase in pork imports under the “Double Epidemics” impact has not had a long-term effect on the Chinese pig industry. But the cost of pork production in China has long been higher than that of significant pork-importing countries such as Europe and the United States. If pork imports continue to grow substantially in the future, it will threaten the pig industry in China. Therefore, China should optimize the structure of the pig trade, to reduce the volatility of the pig industry and realize the stable development of the pig industry. In the strategic requirements of building the domestic cycle as the subject and the domestic and international double cycle, the pig industry in China should fully understand the advantages and disadvantages of domestic and global pig resources, and take the international pig market as an important part of the domestic market while giving full play to the enormous advantages of domestic demand. We will expand the depth and breadth of pork-importing countries, diversify import channels, and avoid trade risks caused by surging imports caused by the outbreak of the epidemic.

The analysis has some limitations. Firstly, we did not consider the substitution of poultry and other meats for domestic pork, mainly due to the following factors: pork import and poultry substitution are different perspectives to make up for the shortage of pork supply in China. The former is from the perspective of the international market, from the perspective of trade vacancies on the pork supply. Therefore, based on the large increase in imported pork after the epidemic outbreak, this paper focuses on the causes of changes in Chinese pork import volume under the ”Double Epidemic,” and whether changes in pork import volume stabilize the domestic market pork supply in the short term. For the latter, from the perspective of the domestic market, after the outbreak of ASF and COVID-19, pork supply in China continues to be short, which affects the pork consumption of Chinese residents, leading most residents to take poultry and other meat as short-term consumption substitutes for domestic pork (24). The data showed that demand for chicken increased by 4.5 percent year-on-year in November 2018. However, in the long run, pork is still the main meat consumed by Chinese residents, and chicken cannot completely replace pork consumption. As Li et al. pointed out, ASF has a more sustainable impact on pork prices, while its impact on chicken prices is short-term (25). At the same time, with the gradual recovery of pig production capacity and the replenishment of imports in the international pork market, the higher replacement share of chicken to pork will be further reduced (26). Therefore, the substitution of poultry and other meats for domestic pork has little influence on this study and is not taken into account in the scope of this study. The relationship between poultry and imported pork is also an area that needs to be further explored in the future. Secondly, in the analysis, due to concerns about sample availability, we used the chilled, fresh, and frozen pork imports as a representative of the data and did not consider breeding pig imports. After the outbreak of ASF, China imported a large number of breeding pigs to restore the number of breeding sows. Considering breeding pigs in the analysis framework can help to analyze the impact of the epidemic on the pig supply capacity from the perspective of the supply chain. Thirdly, to make the research objectives more specific, the data in this paper is from January 2017 to November 2020, which was a relatively short research period. Only the short-term impact of the epidemic was analyzed, and the pork import and pig supply capacity in the post-epidemic period were not analyzed. These are also the directions of future research.

## Conclusion

After the outbreak of ASF and COVID-19, Chinese pork imports showed an upward trend, and the source of pork imports in China increased. Europe, North America, and South America became important sources for Chinese pork imports. The price of domestic pork rose significantly, and the gap between the domestic pork price and the import price was widening. What role pork imports play during the epidemic is uncertain. Therefore, the Armington model was used to analyze the cause of pork imports in China by estimating the change of substitution elasticity between Chinese pork and imported pork in the pre- and post-“Double Epidemics” periods and assess the impact on Chinese pork import demand. Subsequently, this paper explores how pork imports in China alleviate the domestic supply shortage and considers whether the phased differences are affected by seasonal factors. The main conclusions are as follows:

(1) The epidemic changed the elasticity of substitution between domestic pork and imported pork, and the shortage of domestic pork supply became the main reason for the increase in pork imports during the epidemic.

The outbreak of ASF has changed the function of imported pork, leading to an increase in pork imports. Imported pork has been transformed from a complementary product to meet the diversified needs of domestic consumers into a substitute to make up for the pork supply gap. After the outbreak of the COVID-19 epidemic, there is still a gap in the supply of pork in China, and the import demand is enormous. There is a significant substitution relationship between Chinese pork and imported pork. However, with the outbreak of COVID-19 worldwide, pork trade between countries has been hampered, and pork import substitution elasticity in China has decreased. The elasticity of substitution shows that under the impact of the “double epidemic,” the increase of pork import in China makes up for the shortage of pork supply to a certain extent, and will not threaten the development of the pig industry.

(2) During the epidemic period, the supplement of pork imports to the pork supply has dynamic changes.

After the outbreak of the epidemic, the pork supply in China showed dynamic changes. According to the theory of supply and demand, the domestic pork price changed, which led to the lag effect of pork imports in China. Specifically, after the outbreak of ASF, the pork supply in China decreased, and the domestic pork price increased at the end of 2018. After the change of China's live pig transportation policy in China, the supply and demand imbalance between the main producing areas and main selling areas, so the pork import volume increased in January 2019 to make up for the supply gap during the epidemic. In 2020, pork imports increased even after the COVID-19 outbreak in China, as domestic pork prices rose again in August 2019 following a reduction in pig slaughter in China, prompting China to cut temporary tariffs on pork imports in late December 2019. In June 2020, with the increase of breeding sows and pig stocks in China, the fluctuation of domestic pork prices decreased, which also confirmed the empirical results that the elasticity of Chinese pork import demand decreased slightly after the global spread of COVID-19.

## Data availability statement

The original contributions presented in the study are included in the article/supplementary material, further inquiries can be directed to the corresponding author.

## Author contributions

Conceptualization, methodology, software, and writing—original draft preparation: JW. Formal analysis and resources: YC. Data Curation: JW and JZ. Writing—review and editing: JW and GW. Funding acquisition: GW. All authors have read and agreed to the published version of the manuscript.

## Funding

This work was funded by the Natural Science Foundation of Heilongjiang Province in China (Project number LH2019G 002), the Humanities and Social Science Foundation of Ministry of education of China (Project number 21YJA790053), the National Social Science Foundation of China (Project number 22BJY084), and the Chongqing Talents Program in China, any opinion, findings, conclusions, or recommendations expressed in this publication are those of the authors.

## Conflict of interest

The authors declare that the research was conducted in the absence of any commercial or financial relationships that could be construed as a potential conflict of interest.

## Publisher's note

All claims expressed in this article are solely those of the authors and do not necessarily represent those of their affiliated organizations, or those of the publisher, the editors and the reviewers. Any product that may be evaluated in this article, or claim that may be made by its manufacturer, is not guaranteed or endorsed by the publisher.

## Footnotes

^1^From http://www.gov.cn/xinwen/2021zccfh/7/wzsl.htm which is reported by The State Council of the People's Republic of China.

^2^From http://www.gov.cn/xinwen/2019-11/29/content_5457147.htm which is reported by The Chinese government.

^3^From http://zdscxx.moa.gov.cn:8080/nyb/pc/frequency.jsp which is reported by The Ministry of Agriculture and Rural Affairs of China.

^4^According to the classification standard of HS1996, HS0203 is usually called “chill, fresh, frozen pork”. Since pork is imported mainly as frozen, in order to avoid ambiguity, “pork” used uniformly below to represent “chilled, fresh, frozen pork”under the HS code.

^5^From http://zdscxx.moa.gov.cn:8080/nyb/pc/frequency.jsp which is reported by The Ministry of Agriculture and Rural Affairs of China.

^6^The African Swine Fever (ASF) outbreak in China in August 2018 and the COVID-19 outbreak in January 2020 were collectively referred to as “Double Epidemics”.

^7^Armington elasticity was proposed by Armington in 1969 to reflect the substituting between domestic products and imported products.

^8^When calculating the pork import ratio of country (region) i in t period, the formula we usually adopt is as follows: the quantity of pork imported from country (region) i/the total import of pork from China.

^9^Major pork importers include the United States, Canada, Germany, Spain, Brazil, Denmark, France, Netherlands, United Kingdom, Chile, Mexico, Finland and Ireland.

^10^The ratio of pork imports from 13 countries to total pork imports from January to November 2020, according to data from UN comtrade.

^11^From https://data.stats.gov.cn/easyquery.htm?cn=A01&zb=A010301&sj=202105.

^12^Since there is a value of 0, the resulting value is processed by adding 1.

^13^From https://ourworldindata.org/covid-cases.

^14^When the seasonal dummy variable is set, January and February each year, when pork consumption is high, are set to 1, and the other months are set to 0.

^15^Calculation formula:(pork import volume of country i in the current month/pork import volume of country i in the last month)−1.

^16^From http://43.248.49.97/ which is reported by the General Administration of Customs, PRC.
